# Metastatic angiosarcoma of the scalp presented as posttraumatic subgaleal hematoma: The many faces of a diagnostic challenge

**DOI:** 10.1016/j.radcr.2021.06.083

**Published:** 2021-07-23

**Authors:** Loai Aker, Mahir Abdulla Petkar, Sohail Jamiluddin Quazi, Renan Ibrahem Adam

**Affiliations:** a Department of Clinical Imaging, Hamad Medical Corporation, Doha, Qatar; b Department of Laboratory Medicine and Pathology, Hamad Medical Corporation, Doha, Qatar; c Department of Plastic Surgery, Hamad Medical Corporation, Doha, Qatar; d Department of Clinical Imaging, Hamad Medical Corporation, Doha, Qatar

**Keywords:** Angiosarcoma, Head and neck, Lung metastases, Diagnostic radiology

## Abstract

Angiosarcomas represent highly-aggressive malignant lesions of the endothelial cells of blood vessels, affecting mostly the elderly population, and usually located in the scalp or face. As cutaneous angiosarcomas often metastasize to the lung, they can manifest in various forms. We report a case of a 77-year-old male who presented after a posttraumatic blunt scalp lump that was initially diagnosed as infected subgaleal hematoma. This was later found to be an angiosarcoma. Further workup revealed that the tumor was invading the dura, with a rare pattern of mixed concomitant cystic and solid lung metastasis with ground-glass infiltrates. The patient underwent soft tissue reconstruction with split-thickness skin graft for the scalp lesion and palliative chemotherapy. We are discussing the common manifestations of scalp angiosarcomas and their potential pulmonary metastatic patterns. Also, a review of the differential diagnoses that may mimic cutaneous scalp angiosarcoma will be demonstrated.

## Introduction

Angiosarcomas represent a highly-aggressive vascular malignancy affecting the endothelial cells of blood vessels. While angiosarcomas can occur anywhere in the body, they most commonly occur in the face or scalp of elderly population. However, angiosarcomas are rare and represent less than 1% of all head and neck malignancies, and less than 2% of soft tissue sarcomas [1,2]. Cutaneous angiosarcomas rapidly penetrate through the skin, metastasize early, and commonly recur after treatment, resulting in a high-aggressive rate of the tumor. The lung is a common location for angiosarcoma metastases, which may present as pneumothorax, hemothorax, or cystic or solid lesions [1].

## Case summary

**Fig. 1:**
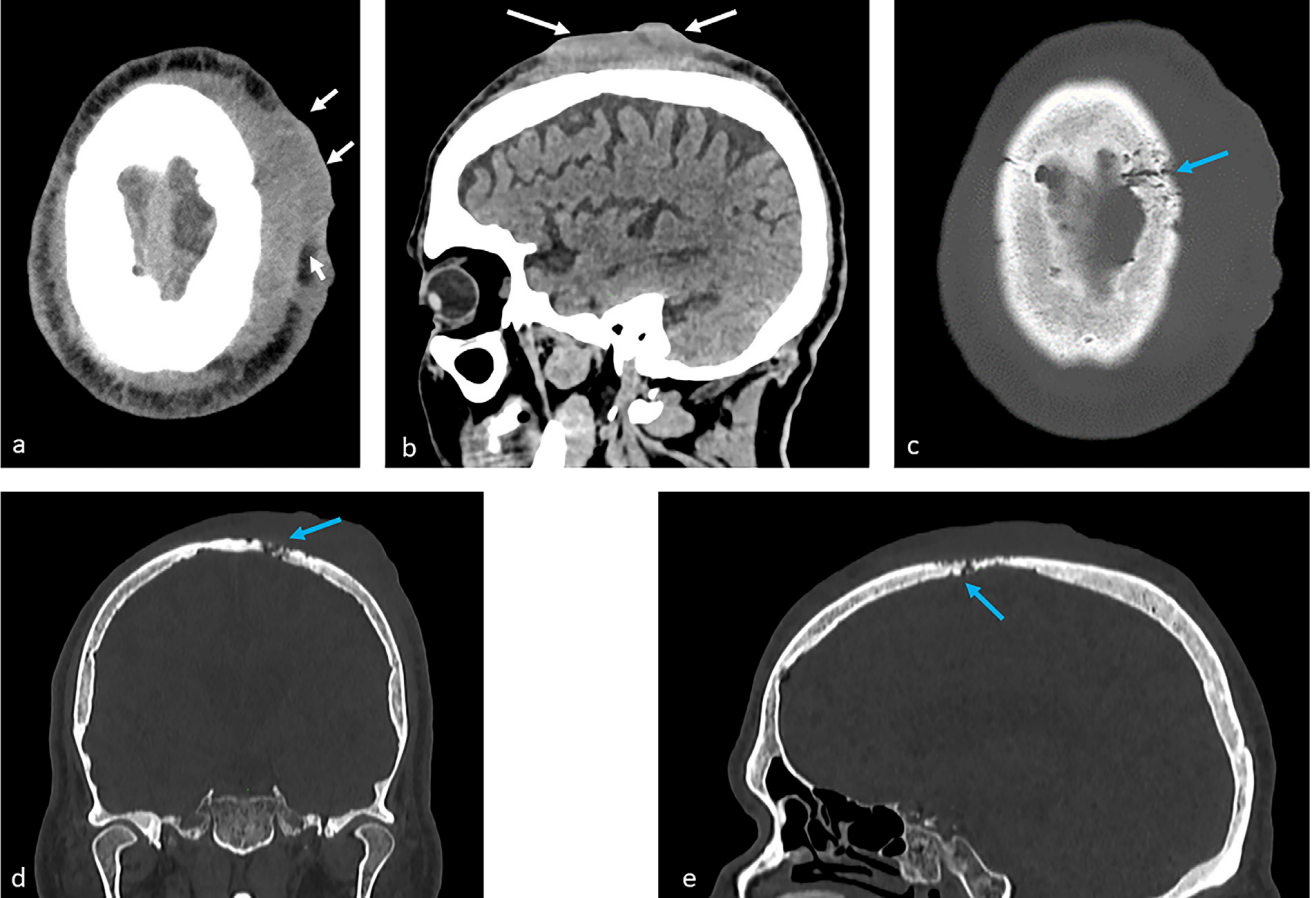
A 77-year-old male with cutaneous angiosarcoma of the scalp. Non–enhanced soft-tissue windowed CT axial (A) and sagittal (B) sections show an irregular exophytic soft tissue lesion arising from the scalp overlying the left frontoparietal bones (white arrows). Bone windowed axial (C), coronal (D) and sagittal (E) sections show associated focal osseous destruction with serpentine osteolytic changes (blue arrows). No intracranial hemorrhage, masses or extension (Color version of the figure is available online.).

A 77-year-old diabetic male presented to the emergency department 1 month after mild blunt head trauma. A soft tissue lump on the left vertex was palpable following the incident. He had associated intermittent mild headache, with no other significant symptoms and with unremarkable neurologic examination. The lump persisted for the next month following the event and on examination, it was soft, erythematous, mildly tender, with slight necrotic changes in the overlying skin. The laboratory findings were unremarkable. Computed tomography (CT) of the head showed no skull fractures or intracranial hemorrhage. A soft tissue scalp thickening and hematoma were appreciated at the left frontoparietal region (Fig. 1). Innumerable stippled and/or serpentine osteolytic foci were present in the underlying left frontoparietal bone. The latter finding was considered involutional bone changes, and the scalp abnormality was misdiagnosed as a non–resolving infected subgaleal hematoma.

The patient was admitted to the hospital, and was planned to undergo debridement. However, the lesion was suspicious intraoperatively as the cutaneous surface of the lesion was variegated, nodular, firm, and ill-defined. Therefore, excisional biopsy for the lesion was done, and confirmation of 5-mm negative margins was obtained. Split-thickness skin graft harvested from the lateral thigh was applied to cover the excised area in the scalp.

**Fig. 2:**
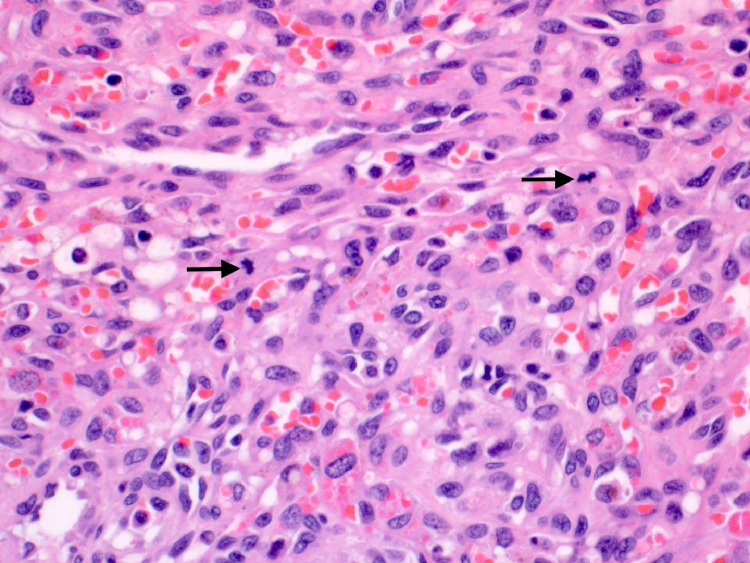
Histopathology image showing anastomosing network of vascular channels, lined by atypical endothelial cells. Note the frequent mitotic activity (black arrows) (H and E x 40).

Histopathological examination of the specimen revealed an infiltrating neoplasm composed of anastomosing vascular channels lined by atypical endothelial cells, displaying frequent mitosis (Fig. 2). The lesion was positive for various vascular markers including CD31, CD34, erg, Fli-1 and D2-40. Cytokeratins (MNF 116 and CK AE1/AE3) and HHV immunostains were negative. Ki-67 showed a high proliferative index of approximately 40%. The morphology and immunohistochemical features were in keeping with Angiosarcoma, Grade 2 (French Federation of Cancer Centers Sarcoma Group [FNCLCC] histologic grading).

**Fig. 3:**
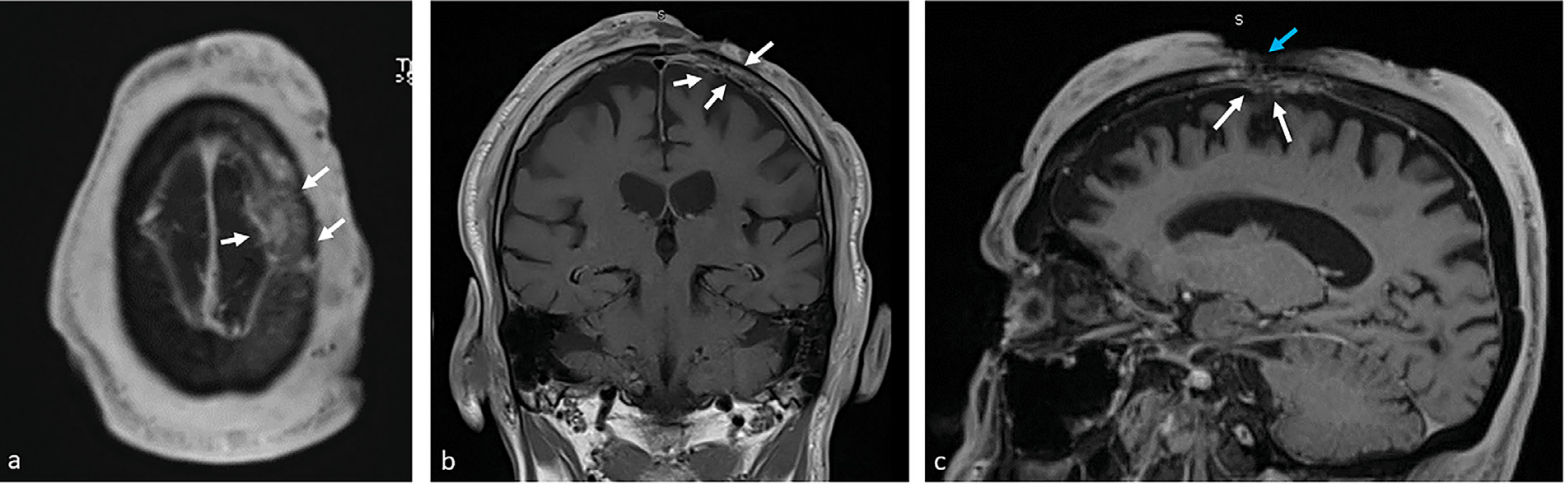
MRI head of the patient with cutaneous angiosarcoma of the scalp after local excision of the lesion. Axial (A) and coronal (B) post gadolinium contrast T1-weighted images, and sagittal (C) MPRAGE image demonstrate residual part of the lesion that is infiltrating the dura and causing dural enhancement (white arrows), and infiltrating the left frontal and parietal bone (blue arrow). Findings indicate local invasion of the tumor (Color version of the figure is available online.).

Further work-up for metastatic evaluation included head MRI, and CT scan of the neck, chest, abdomen, and pelvis. Head MRI showed that the lesion was infiltrating the left frontal and parietal bones, passing through the calvarium and infiltrating the dura with evidence of dural enhancement (Fig. 3).

**Fig. 4:**
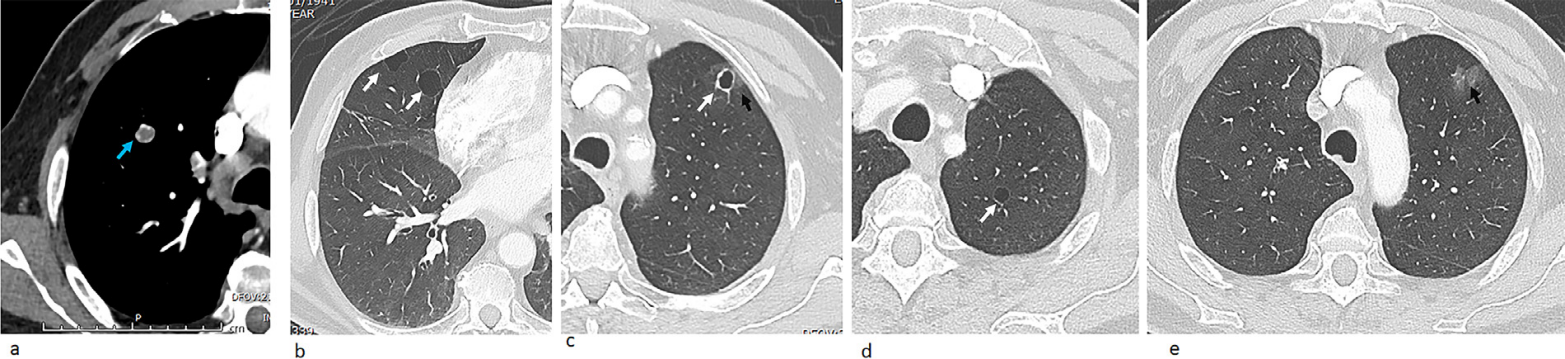
An 80-year-old male with a metastatic angiosarcoma of the scalp. Contrast enhanced axial (A-E) sections of lung CT scan showed multiple thin-walled cysts (white arrows) accompanied with ground-glass attenuation which are likely marking areas of hemorrhage (black arrows). A solid pulmonary nodule with stippled calcifications and enhancing rims is noted in the right upper lung lobe (blue arrow). These lesions represent pulmonary metastatic deposits of the scalp angiosarcoma (Color version of the figure is available online.).

Neck CT revealed subcentimetric lymph nodes that appeared suspicious for malignancy due to their rounded shape, and loss of fatty hilum. Interestingly, chest CT revealed multiple pulmonary thin-walled cysts, a cavitary lesion with peripheral calcifications and/or rim enhancement, scattered regions of ground glass opacities, and a solid pulmonary nodule with stippled calcifications and enhancing rims (Fig. 4).

Meanwhile, the surgery site was not healing properly, with residual nodular lesions and tendency to bleed after light touch which required blood transfusions. Palliative chemotherapy of paclitaxel was started. The patient expired in 3 months’ after initial presentation.

## Discussion

Angiosarcomas represent a highly-aggressive vascular malignancy that originate from the endothelial cells of vascular or lymphatic structures. While they can occur anywhere in the body, they most commonly affect the face or scalp of elderly patients. Angiosarcomas are rare and represent less than 1% of all head and neck malignancies, and less than 2% of soft tissue sarcomas [1,2]. Cutaneous angiosarcomas commonly manifest as dark-purple, bluish or red nodular or plaque-like lesions. These lesions may undergo ulceration or bleeding after minor manipulation like hair combing [3]. This was evident in our case after surgical intervention to initially debride it, before the suspicion of malignancy. In the early stages, the lesions can mimic other entities like cellulitis, infection or skin injuries [4]. These lesions can be overlooked clinically due to their vague cutaneous manifestation and potential obscuration by the scalp hair. The clinical and radiologic teams should be suspicious of angiosarcomas in elderly patients presenting with an enlarging lump in the head or neck, with manifestations of anemia or coagulopathy. Also, some risk factors can be alarming, like previous history of chronic lymphedema, radiation therapy, Maffucia syndrome, and neurofibromatosis type 1 [5]. Other potential early dermatologic presentations may include an enlarging bluish or violaceous bruise, a discolored nodule that may bleed, or non–resolving ulceration. Extensive local growth and multifocality are common and might represent a late stage of the disease with a worse prognosis [6]. In our case, the patient had atypical presentation of mild head trauma followed by non–resolving scalp lump, that was initially considered an infected subgaleal hematoma. Therefore, clinicians should have a high level of suspicion for the disease to allow for early diagnosis. The differential diagnosis of scalp lesions includes hemangioblastoma, metastatic lesion from another primary location, sinonasal squamous-cell cancer, Kaposi sarcoma, and Merkel cell carcinoma [7]. Primary brain angiosarcoma is very rare, with only isolated cases reported in the literature [4].

When scalp angiosarcoma is suspected clinically, head CT scan is recommended as the best initial imaging modality to characterize bone alterations that might be related to the lesion. MRI is an essential modality for evaluating the lesion extension for both the skin and intracranial spaces [8]. Generally, radiologic evaluation for suspicious dermatologic lesions is recommended for the assessment of possible bony invasion, orbital affection, perinerual spread, and the extent of tumor invasion in soft tissue and for staging of lymph nodes and metastatic disease [9]. MRI findings of scalp angiosaroma include high T2-weighted intensity signal and intermediate signal on T1-weighted images with areas of hyperintense signal corresponding to hemorrhagic foci. The most distinguishing feature is the presence of low signal intensities on both T1 and T2 weighted images which represent high-flow serpentine vessels in a solid soft-tissue mass. Though, high signal in T2 weighted images may indicate low flow vessels. Post-contrast images show areas of enhancement and may show non–enhancing areas that reflect tumor necrosis. The latter feature may be non–specific but can be reflect the fast growth of a malignant lesion. Therefore, some investigators suggested that irregular lesion margins, heterogeneous signal, and large size are indicators of a soft tissue malignant lesion [10]. Unless a distinguishing finding of angiosarcoma can be detected, indeterminate lesions should raise the suspicion of potential malignancy, and necessitate further workup and biopsy [4]. Generally, T2-weighted images with the fat saturation and postcontrast T1-weighted images with the fat saturation techniques were found helpful in visualizing the whole extent of angiosarcoma through the scalp layers and the skull, which might not be revealed in clinical examination. These MRI findings are important for further therapeutic planning for patients with scalp angiosarcoma [11].

Cutaneous angiosarcomas rapidly penetrate through the skin, early metastasize mainly hematogenously and commonly recur after treatment. The lung is the most common location for angiosarcoma metastases. Liver, bone and lymph nodes are other common sites [12]. Pulmonary metastases from angiosarcomas may appear as bilateral peripheral solid nodules in chest radiographs. However, it is difficult to differentiate them from other metastatic tumors. Common CT features include multiple solid nodules, followed by thin-walled lung cysts. Many solid lesions demonstrate inhomogeneous contrast enhancement. Also, mixed pattern of concomitant solid and cystic tumors have been reported. Other potential CT findings include pneumothorax, pleural effusion, hilar adenopathy, and punctate calcification [1,[13], [14], [15]]. Hemorrhagic changes in either the cystic or solid lesions were described as a characteristic feature of metastatic angiosarcomas. These hemorrhages occur due to the fragility of neovascular tissues, and appear as air-fluid levels within the lung cysts, hemothorax, diffuse pulmonary infiltrations, or as ground-glass attenuations [4,15]. Our case is unique as it demonstrates the concomitant mixed pattern of solid and cystic lung lesions, with associated ground-glass opacities.

In a large series of 434 cutaneous angiosarcomas cases with over 34 years of surveillance, Albores-Saavedra *et al.* reported that the mean age for patients was 73 years, and the primary location of most cutaneous angiosarcoma lesions was in the head and neck [16]. A lower survival rates was associated with older groups; with a 10-year survival rate of 72% in patients younger than 50 years, and 37 % in patients older than 50 years. Interestingly, lesions arising from the head, and neck had a 14% worse 10-year survival rate [16].

In another series, very poor prognosis was reported for angiosarcoma tumors that metastasize, with a 4-month survival for cases with lung metastases [17]. This is concordant with our case, as the survival of the patient from the time of diagnosis was less than 3 months.

Different treatment modalities were reported in the literature. Thus, effective treatment of angiosarcoma is not clearly established [1]. Wide surgical excision of the lesion was suggested as the mainstay of treatment in an attempt to achieve tumor-free margins, in addition to adjuvant radiotherapy [1,^2^,17]. In cases with non–operable multifocal and widespread lesions, radiation therapy was suggested. Yet, the reported results were suboptimal [1,17]. Other treatment options utilizing chemotherapy agents like paclitaxel were tried, especially with unresectable lesions [1,18], but with limited reported success.

## Conclusion

Angiosarcoma is a rare aggressive tumor with early metastases. The cutaneous form is the most common, and most cases occur in the head and neck, and typically after 60 years of age. Scalp angiosarcoma can have different dermatologic manifestation that include enlarging painful lump, or a non–resolving bruise. Metastases to cervical lymph nodes and the lungs are common. We are reporting an atypical clinical presentation with a rare pattern of mixed concomitant cystic and solid lung metastasis with ground-glass infiltrates. While multimodality imaging is vital for proper staging and management, it is important for clinicians and radiologists to be oriented about the potential radiological findings and correlate them with suspicious clinical findings and risk factors to achieve early diagnosis of these challenging malignancies.

## Ethical approval

All procedures performed in the studies involving human participants were in accordance with the ethical standards of the institutional and/or national research committee and with the 1964 Helsinki Declaration and its later amendments or comparable ethical standards.

This article does not contain any studies with animals performed by any of the authors.

## Patient consent

Written informed consent was obtained from the patient for publication of this case report and the accompanying images. A copy of the written consent is available for review by the Editor-in-Chief of this journal on request.

## Declarations

Ethics approval: An approval from Hamad Medical Corporation, Medical Research Council (MRC) was obtained prior to submission of this manuscript.

Funding statement: This article did not receive any specific grant from funding agencies in the public, commercial, or not-for-profit sectors.
